# Supplementary material for: “Yellow” laccase from Sclerotinia sclerotiorum is a blue laccase that enhances its substrate affinity by forming a reversible tyrosyl-product adduct
Source: PLoS One. 2020 Jan 21;15(1):e0225530. doi: 10.1371/journal.pone.0225530 (PMC6974248; doi:10.1371/journal.pone.0225530)
Supplement: S1 Fig — UV-vis spectra of blue laccase (black), blue laccase after turnover with excess of dimethanolferrocene (red) and 2,3-dimethoxy-6-methyl-1,4-benzoquinone (blue) The substrates with no radical forming ability or with extremely short life time do not form adducts in in the same reaction time scaling. (DOCX) [file pone.0225530.s001.docx]

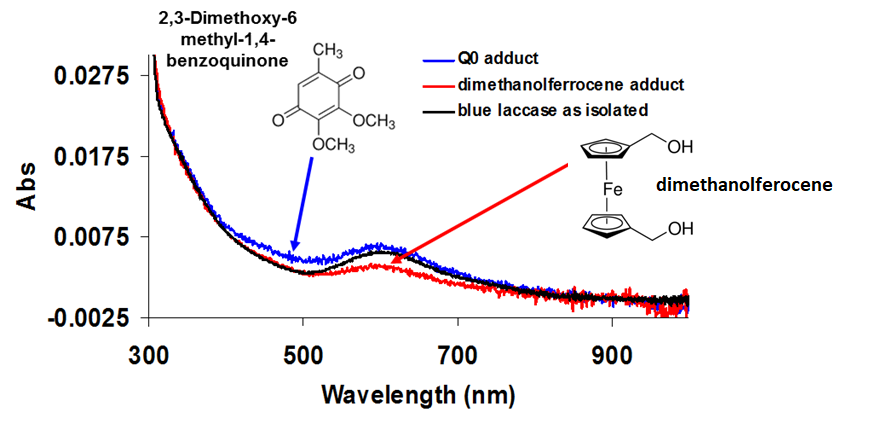


**S1 Fig.** **UV-vis spectra of laccase after turnover with substrates with no radical forming ability.** UV-vis spectra of blue laccase (black), blue laccase after turnover with excess of dimethanolferrocene (red) and 2,3-dimethoxy-6-methyl-1,4-benzoquinone (blue) The substrates with no radical forming ability or with extremely short life time do not form adducts in in the same reaction time scaling.
