# Supplementary material for: “Yellow” laccase from Sclerotinia sclerotiorum is a blue laccase that enhances its substrate affinity by forming a reversible tyrosyl-product adduct
Source: PLoS One. 2020 Jan 21;15(1):e0225530. doi: 10.1371/journal.pone.0225530 (PMC6974248; doi:10.1371/journal.pone.0225530)
Supplement: S2 Fig — UV-vis spectra of nitrated laccase and ABTS-nitrated laccase compared to the normal ABTS-laccase adduct. (DOCX) [file pone.0225530.s002.docx]

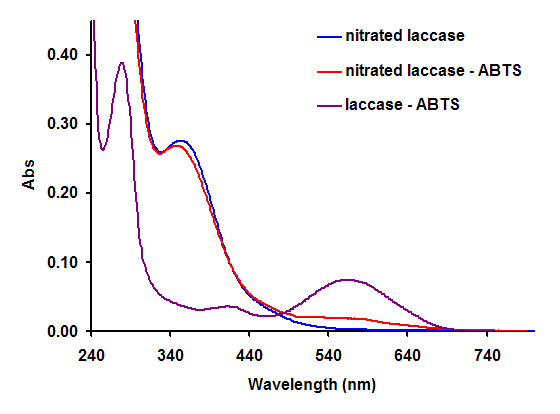


**S2 Fig**. **Effect of nitration upon laccase adduct formation.** UV-vis spectra of nitrated laccase and ABTS-nitrated laccase compared to the normal ABTS-laccase adduct.
