# Supplementary material for: “Yellow” laccase from Sclerotinia sclerotiorum is a blue laccase that enhances its substrate affinity by forming a reversible tyrosyl-product adduct
Source: PLoS One. 2020 Jan 21;15(1):e0225530. doi: 10.1371/journal.pone.0225530 (PMC6974248; doi:10.1371/journal.pone.0225530)
Supplement: S3 Fig — ESI(+)-HRMS spectrum of the peptide that is missing in the tryptic digestion of ABTS-laccase adduct but is observed in the blue laccase digestion. (DOCX) [file pone.0225530.s003.docx]

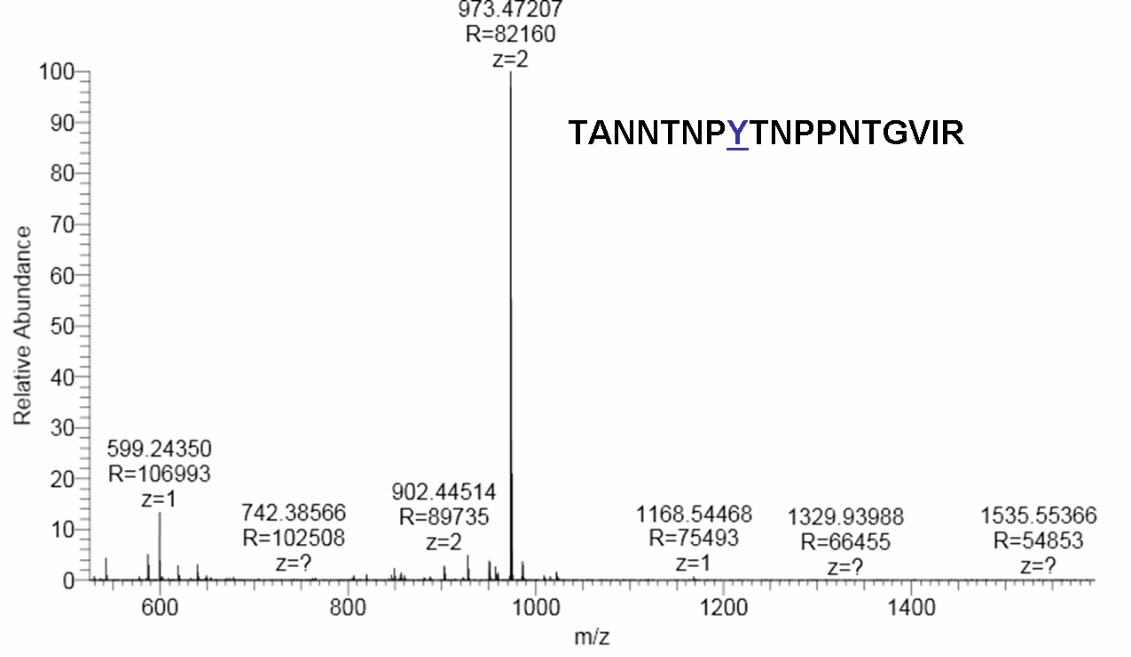


**S3 Fig.** **Identification via MS of Tyr responsible for adduct formation.** ESI(+)-HRMS spectrum of the peptide that is missing in the tryptic digestion of ABTS-laccase adduct but is observed in the blue laccase digestion.
