# Supplementary material for: “Yellow” laccase from Sclerotinia sclerotiorum is a blue laccase that enhances its substrate affinity by forming a reversible tyrosyl-product adduct
Source: PLoS One. 2020 Jan 21;15(1):e0225530. doi: 10.1371/journal.pone.0225530 (PMC6974248; doi:10.1371/journal.pone.0225530)
Supplement: S4 Fig — The structures of the three identified compounds are shown in left top panel. (DOCX) [file pone.0225530.s004.docx]

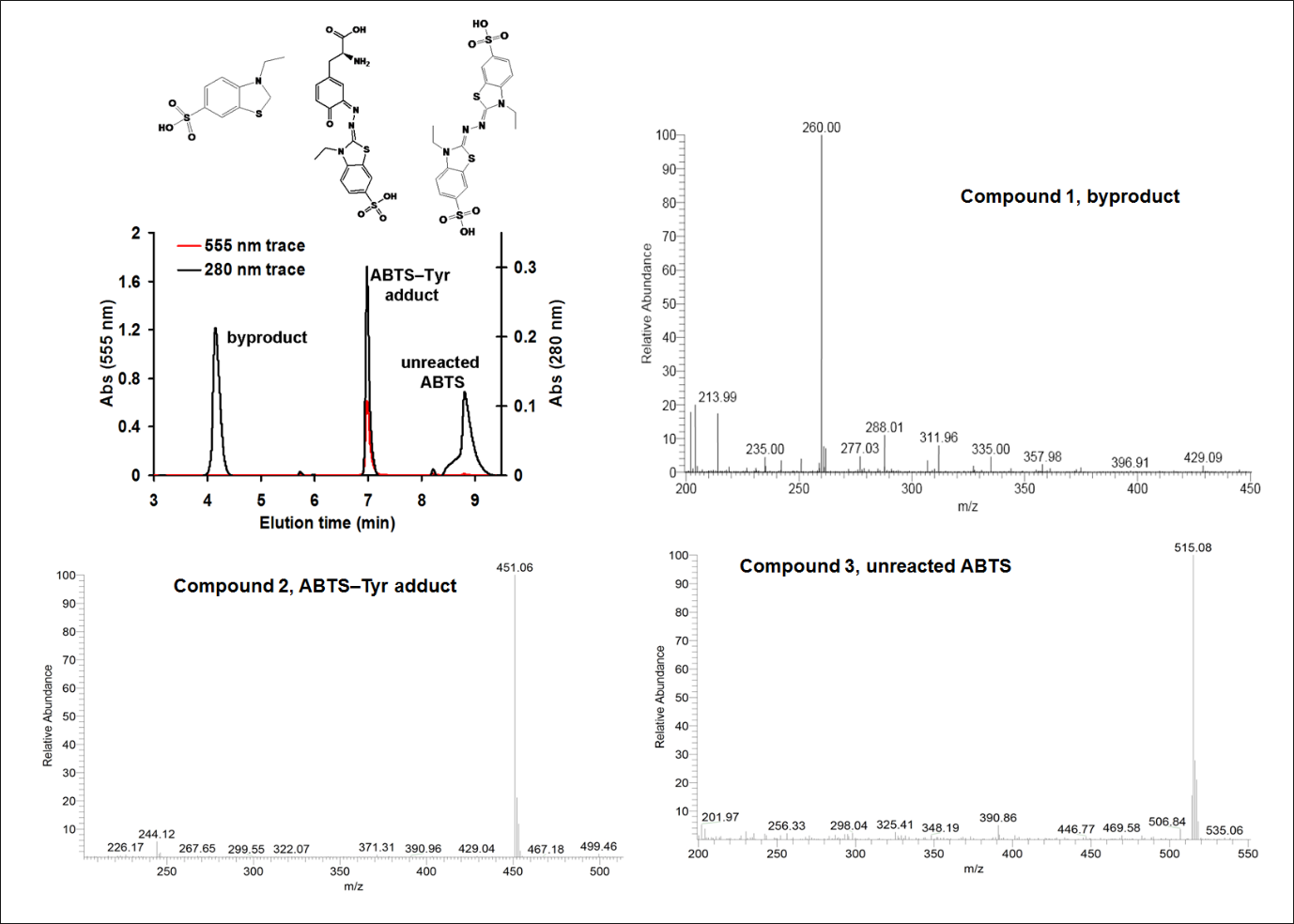


**S4 Fig.** **HPLC-MS separation of a tyrosine-ABTS reaction mixture and identification**

**of the reaction products.** The structures of the three identified compounds are shown in left top panel.
