# Supplementary material for: “Yellow” laccase from Sclerotinia sclerotiorum is a blue laccase that enhances its substrate affinity by forming a reversible tyrosyl-product adduct
Source: PLoS One. 2020 Jan 21;15(1):e0225530. doi: 10.1371/journal.pone.0225530 (PMC6974248; doi:10.1371/journal.pone.0225530)
Supplement: S5 Fig — (DOCX) [file pone.0225530.s005.docx]

**S5 Fig.** **UV-vis spectra of the free Tyr-ABTS adduct and byproduct.**
