# Supplementary material for: “Yellow” laccase from Sclerotinia sclerotiorum is a blue laccase that enhances its substrate affinity by forming a reversible tyrosyl-product adduct
Source: PLoS One. 2020 Jan 21;15(1):e0225530. doi: 10.1371/journal.pone.0225530 (PMC6974248; doi:10.1371/journal.pone.0225530)
Supplement: S6 Fig — Michaelis-Menten curves for blue (A), yellow (B), ABTS (C), guaiacol (D), TMB (E) laccases and their Eadie-Hofstee linearization plots (as insets) using Q0H2 as substrate. KM and kcat values are listed in Table 1 from the main manuscript. (DOCX) [file pone.0225530.s006.docx]

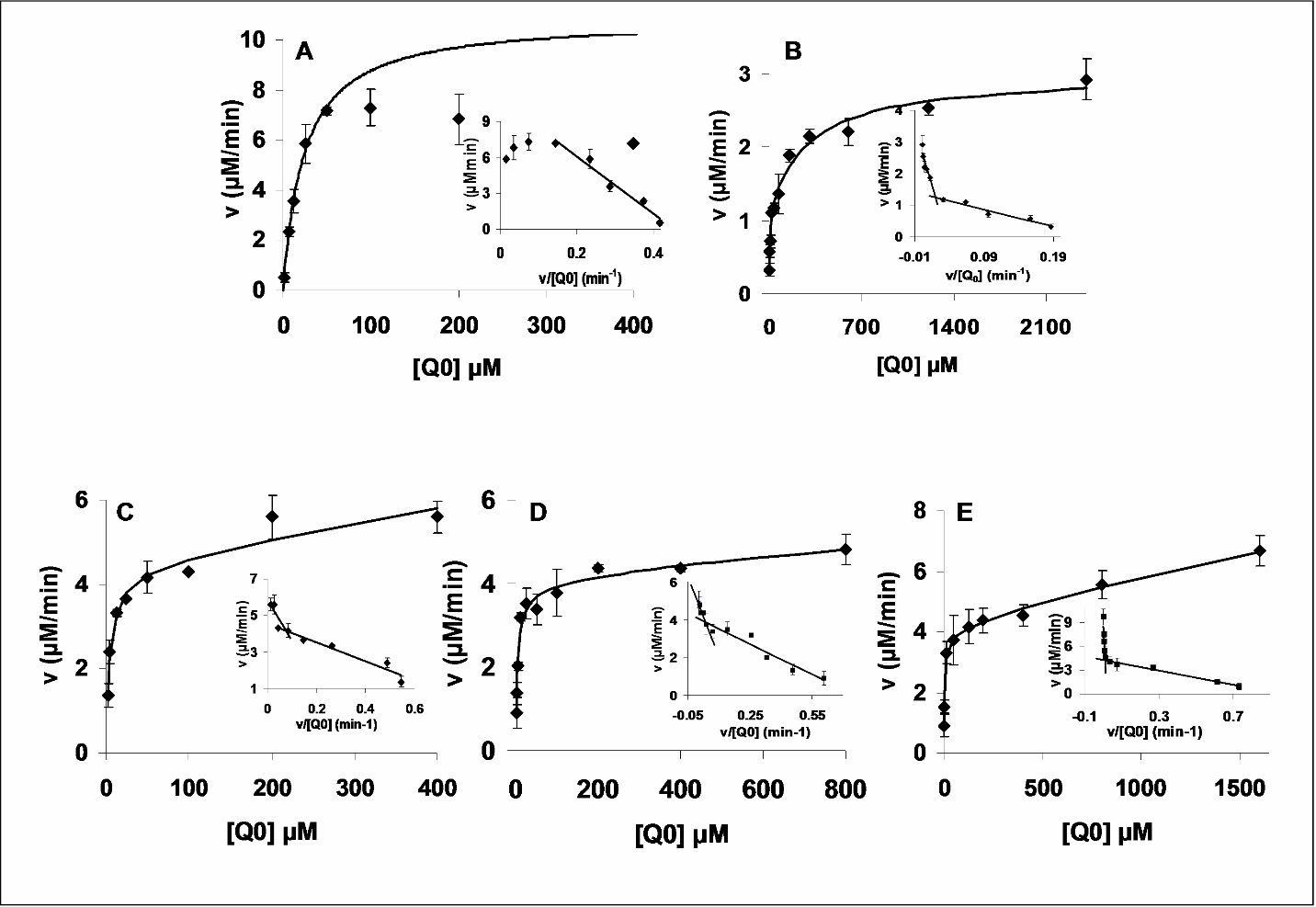


**S6 Fig.** **Enzymatic kinetic investigations for the studied laccase adducts.** Michaelis-Menten curves for blue (A), yellow (B), ABTS (C), guaiacol (D), TMB (E) laccases and their Eadie-Hofstee linearization plots (as insets) using Q_0_H_2_ as substrate. K_M_ and k_cat_ values are listed in Table 1 from the main manuscript.
