# Supplementary material for: “Yellow” laccase from Sclerotinia sclerotiorum is a blue laccase that enhances its substrate affinity by forming a reversible tyrosyl-product adduct
Source: PLoS One. 2020 Jan 21;15(1):e0225530. doi: 10.1371/journal.pone.0225530 (PMC6974248; doi:10.1371/journal.pone.0225530)
Supplement: S7 Fig — A. Modelled 3D structures of the S. sclerotinia laccase. Model of S. sclerotiorum laccase using the better-known ascomycete laccases as template (pdb code 2Q9O, Melanocarpus albomyces). Copper ions are depicted as orange spheres. Histidines involved in the trinuclear site are colored in yellow and the key tyrosine (Y23) identified by MS data is colored in purple (see main text). Notice the key position of the tyrosine between the three domains of the enzyme and just above the channel from the T2/T3 site. B. Same model with all Tyr residues indicated. (DOCX) [file pone.0225530.s007.docx]

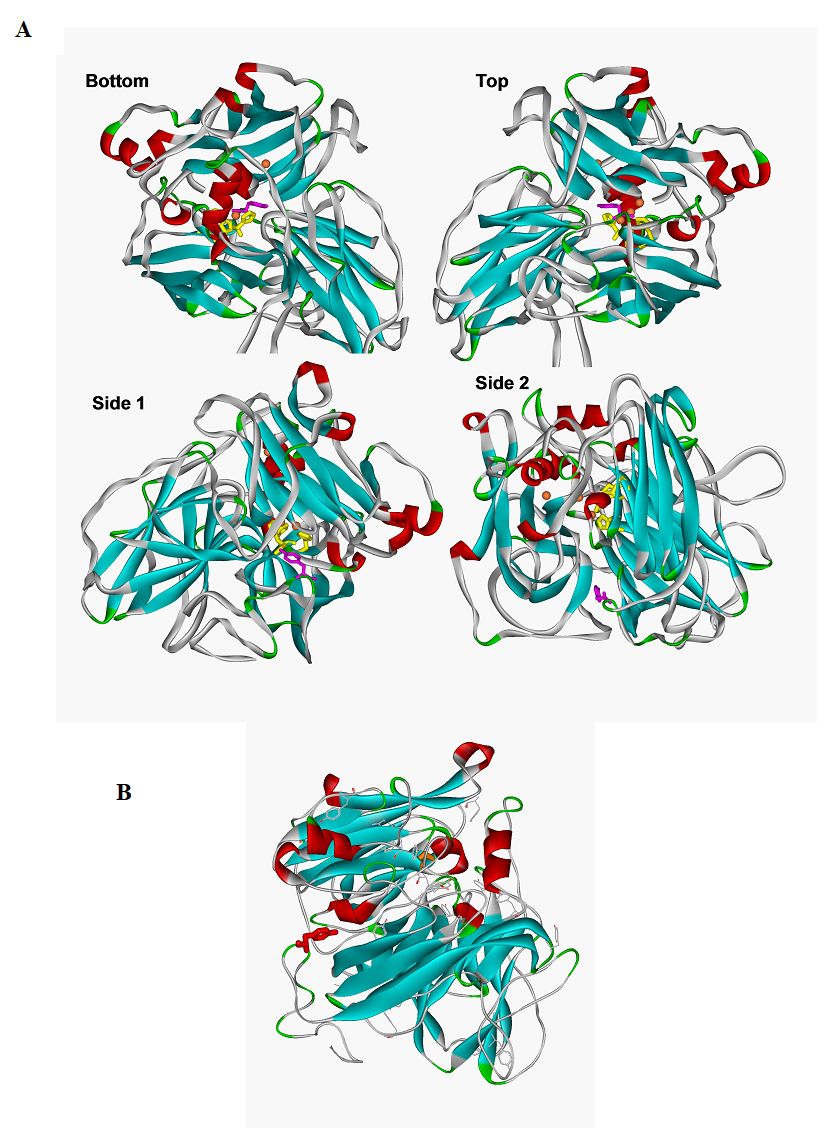


**S7 Fig.** **A.** **Modelled 3D structures of the S. sclerotinia laccase.** Model of *S. sclerotiorum* laccase using the better-known ascomycete laccases as template (pdb code 2Q9O, *Melanocarpus albomyces*). Copper ions are depicted as orange spheres. Histidines involved in the trinuclear site are colored in yellow and the key tyrosine (Y23) identified by MS data is colored in purple (see main text). Notice the key position of the tyrosine between the three domains of the enzyme and just above the channel from the T2/T3 site. **B.** Same model with all Tyr residues indicated.
