# Supplementary material for: “Yellow” laccase from Sclerotinia sclerotiorum is a blue laccase that enhances its substrate affinity by forming a reversible tyrosyl-product adduct
Source: PLoS One. 2020 Jan 21;15(1):e0225530. doi: 10.1371/journal.pone.0225530 (PMC6974248; doi:10.1371/journal.pone.0225530)
Supplement: S8 Fig — The structure was modeled having Melanocarpus albomyces laccase as template (residue numbering is from the uniprot database entry A7EM18). (DOCX) [file pone.0225530.s008.docx]

**
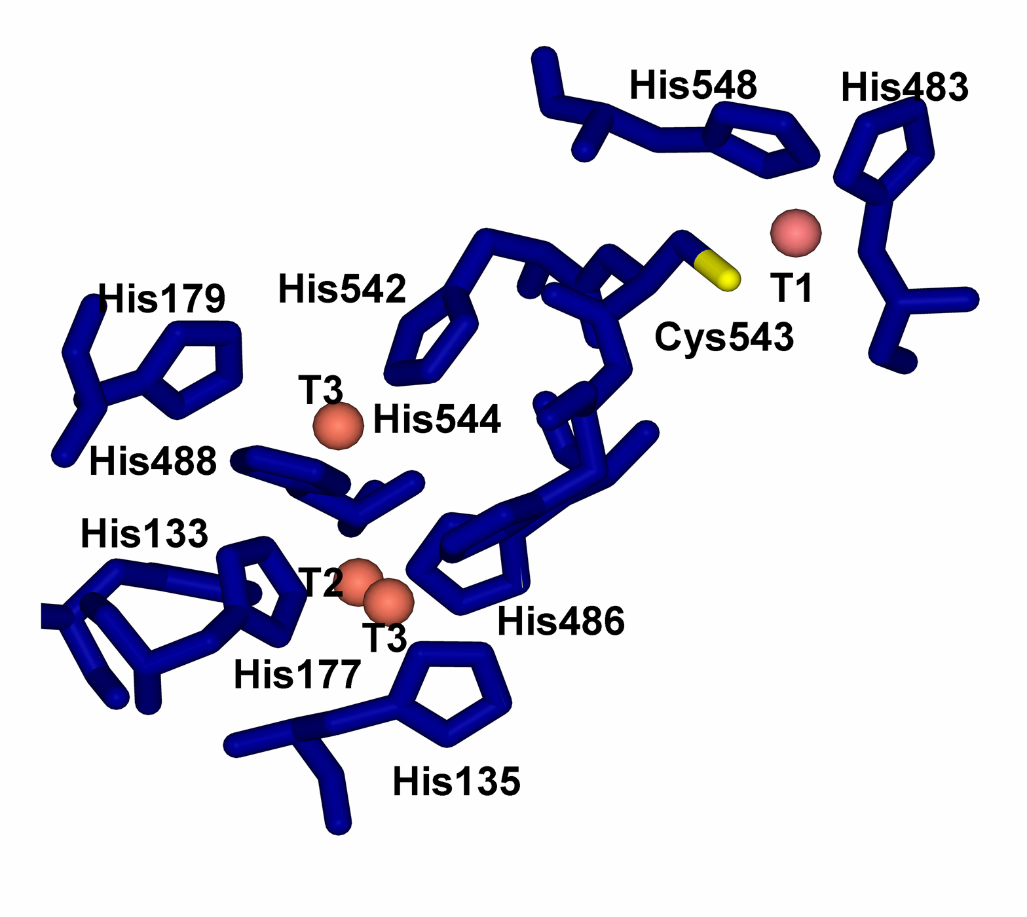
**

**S8 Fig.** **Modeled active sites of *S. sclerotiorum* laccase.** The structure was modeled having *Melanocarpus albomyces* laccase as template (residue numbering is from the uniprot database entry A7EM18).
